# Centromere Protein (CENP)-W Interacts with Heterogeneous Nuclear Ribonucleoprotein (hnRNP) U and May Contribute to Kinetochore-Microtubule Attachment in Mitotic Cells

**DOI:** 10.1371/journal.pone.0149127

**Published:** 2016-02-16

**Authors:** Younghwa Chun, Raehyung Kim, Soojin Lee

**Affiliations:** Department of Microbiology and Molecular Biology, College of Bioscience and Biotechnology, Chungnam National University, Daejeon, Republic of Korea; Northwestern Medical Faculty Foundation, UNITED STATES

## Abstract

**Background:**

Recent studies have shown that heterogeneous nuclear ribonucleoprotein U (hnRNP U), a component of the hnRNP complex, contributes to stabilize the kinetochore-microtubule interaction during mitosis. CENP-W was identified as an inner centromere component that plays crucial roles in the formation of a functional kinetochore complex.

**Results:**

We report that hnRNP U interacts with CENP-W, and the interaction between hnRNP U and CENP-W mutually increased each other’s protein stability by inhibiting the proteasome-mediated degradation. Further, their co-localization was observed chiefly in the nuclear matrix region and at the microtubule-kinetochore interface during interphase and mitosis, respectively. Both microtubule-stabilizing and microtubule-destabilizing agents significantly decreased the protein stability of CENP-W. Furthermore, loss of microtubules and defects in microtubule organization were observed in CENP-W-depleted cells.

**Conclusion:**

Our data imply that CENP-W plays an important role in the attachment and interaction between microtubules and kinetochore during mitosis.

## Introduction

Kinetochores are DNA-protein multicomplexes that are central to accurate separation of genetic information during mitosis [1]. Their primary duty is to provide a landing pad for microtubules, holding them faithfully until the duplicated chromosomes reach their respective poles in the cell [2]. Proper interplay between kinetochores and microtubules is, therefore, the most salient aspect of kinetochore function during mitosis. Deregulation of this function is highly associated with abnormalities like cancer in humans [3]. Microtubule dynamic instability is often used to describe the metastable nature of microtubule polymers [4]. How these highly dynamic mitotic spindles are stably anchored to kinetochores, and how the latter communicate with microtubules are yet unresolved.

Heterogeneous nuclear ribonucleoprotein (hnRNP) U is an abundant nuclear protein and a component of hnRNP complex, which binds to nascent hnRNA [5]. The same protein was also named as scaffold attachment protein A (SAF-A), thought to selectively bind to scaffold/matrix attached region (SAR/MAR) sequences within the genome where nuclear matrix attaches [6]. This multifaceted protein was later identified to function in various crucial activities in the nucleus, such as the recruitment of *XIST* RNA in inactive X chromosome [7], and modulation of heterochromatin protein 1α (HP1α) activity [8]. Furthermore, Ma *et al*. recently identified hnRNP U as a microtubule-associated protein [9]. They observed hnRNP U to be localized at the mitotic spindles during mitosis and found that the stability of kinetochore-microtubule attachment was significantly reduced in hnRNP U-depleted cells [9].

CENP-W was originally discovered as cancer-upregulated gene 2 (CUG2) [10]. As the name indicates, this gene is commonly overexpressed in several tumor types [10]. Later, CENP-W was identified as a member of the inner kinetochore plate, forming a complex with CENP-T [11, 12]. Depletion of the CENP-W/CENP-T complex led to severely defective recruitment of other kinetochore proteins, indicating that the complex functions upstream to these components during functional kinetochore formation [11]. Concordantly, CENP-W-depleted cells presented frequent mitotic abnormalities such as chromosome misalignment and multipolar figures [12]. Previously, we reported that CENP-W localizes in nucleoli, enhancing stability of the pre-kinetochore complex, which is required for proper recruitment of kinetochore components during mitotic prophase [13, 14]. In this study, we show that CENP-W interacts with hnRNP U, and participates in the kinetochore-microtubule interaction and during mitosis.

## Materials and Methods

### Cells, Plasmids, and siRNAs

293T cells [10] and HeLa-FLAG-CENP-W stable cells [12] were cultured in Dulbecco's Modified Eagle Medium supplemented with 10% fetal bovine serum under the standard cell culture condition. Transient transfection of was performed using either Effectene^™^ (Qiagen) or polyethylenimine reagent (PEI, Sigma). Plasmids expressing FLAG-CENP-W and glutathione *S*-transferase (GST)-CENP-W were described previously [12]. The cDNA clones of human hnRNP U was purchased from 21C frontier human gene bank (Daejeon, Korea), was subcloned into pKH3-3HA plasmid (a gift from Dr. Changhoon Kim, Korea Basic Science Institute, Korea) using EcoRI-KpnI. For GST-fusion protein, hnRNP U were inserted into the pEBG vector [12] using BamHI-KpnI. The cDNA gene of human SPC24 and SPC25 were obtained by PCR-based amplification using isolated HeLa total RNAs and inserted into pKH3-3HA vector using BamHI and EcoRI sites. To generate histidine-tagged recombinant proteins, coding region of 3HA-SPC24 or 3HA-SPC25 was subcloned into the pET28a(+) vector (Novagen). For knockdown experiment, siRNAs synthesized from Bioneer (Korea) were transfected to cells with Lipofectamin^™^ (Invitrogen). The siRNA sequences were as follows. CENP-W: CAGAUAAAGCGGAAGGCUC, hnRNP U: AAAGACCACGAGAAGAUCAUG, and siGAK: GCGACACGGUUCUGAAGAU.

### Protein binding assays, and antibodies

Co-immunoprecipitation and GST pulldown were performed as previously described [13]. Briefly, cell lysates were incubated with 1 μg of either the specific antibody or preimmune mouse IgG (Millipore) for 2 h at 4°C. For GST pulldown assay, the lysates were incubated with 20 μL of a 50% slurry suspension of glutathione agarose beads (GE Healthcare).

Mouse monoclonal anti-FLAG (F1804) and rabbit polyclonal anti-MYC (C3956) antibodies were purchased from Sigma-Aldrich, and rabbit polyclonal anti-his (sc-803), mouse monoclonal anti-HA (sc-7392), mouse monoclonal anti-GFP (sc-9996), rabbit polyclonal anti-CENP-A (sc-22787), rabbit polyclonal anti-CENP-B (sc-22788), and mouse monoclonal anti-hnRNP U (sc-32315) antibodies from Santa Cruz Biotechnology. Rabbit polyclonal anti-CENP-W antibody was obtained from Abnova (PAB25656, Taiwan).

### Nuclear matrix isolation and fluorescence microscopy

Nuclear matrix was isolated based on the high-salt isolation method described previously [13]. Briefly, following incubation of cells in CSK buffer containing 0.5% Triton X-100 for 5 min at 4°C, the insoluble pellet was fractionated by centrifugation. Next, the chromatin fraction was obtained by DNase I (1 unit/μL) treatment and subsequent ammonium sulfate (0.25M) extraction. After washing with CSK buffer containing 2 M NaCl, the remaining pellet (nuclear matrix fraction) was dissolved with a buffer containing 8 M urea.

For the fluorescence microscopy, cells were fixed with 4% paraformaldehyde in phosphate-buffered saline (PBS) for 15 min, followed by permeabilization with 0.1% Triton X-100 in PBS. Then, cells were incubated with appropriate primary antibody for 1 h, followed by addition of either fluorescein isothiocyanate (FITC)-labeled anti-mouse (Vector Laboratories, FI-2000) or cyanine (Cy)3-linked anti-rabbit (Jackson ImmunoResearch, 711-165-152) antibodies. When necessary, cells were synchronized by treatment with either double thymidine block (2 mM, two 16-h treatment with a 10-h interval) or nocodazole (100 ng/mL) treatment for 12 h. For in situ microscopy, cells were fixed and incubated with either DNase I (1 unit/μL) or RNase A (200 μg/mL) at 30°C for 20 min prior to antibody treatment [13]. Imaging was performed using an Olympus IX70 fluorescence microscope at 200 × magnification.

### Glycerol gradient fractionation and gel filtration chromatography

For cell fractionation, cell lysates in a buffer (50 mm Tris-HCl, pH 8.0; 120 mm NaCl; 5 mm EDTA; and 1% NP-40) was applied onto the 10–40% linear glycerol gradient, followed by centrifugation at 40,000 rpm in a Beckman SW 41 Ti rotor for 20 h at 4°C. Fractions of 1 mL volume were collected, and the first fraction from the bottom was labeled as fraction 1.

For gel filtration chromatography, the precleared HeLa-CENP-W lysates were loaded onto Sephacryl S-300 HR column (GE healthcare, 1.6 x 40 cm) equilibrated in a buffer (50 mM sodium phosphate, 150 mM NaCl, pH 7.2). Fractions of 1 ml were collected.

### Cold-stable microtubule stability and microtubule regrowth assay

The cold-mediated microtubule depolymerization assay was performed as described previously with some modifications [9]. HeLa-CENP-W cells cultured on 0.1% gelatin-coated coverslips were incubated at 4°C for 10 min prior to fixation. Next, the cells were incubated with 10% casblock (Invitrogen) in PBS for 30 min prior to antibody treatment. For microtubule regrowth assay, cells were placed at 0°C for 30 min, after which microtubules were allowed to regrowth by incubating the cells at 37°C for 10 min.

Kinetochore-derived microtubules were monitored according to a protocol reported previously [15]. Forty-eight hours post siRNA transfection, the HeLa cells were further incubated with nocodazole (100 ng/mL) for 6 h. After thorough washing, the cells were fixed at 1, 7, or 15 min time-points after media change. The samples were then immunostained with mouse monoclonal anti-α-tubulin (Sigma-Aldrich, T6199) and anti-centromere antibody (ACA) (Antibodies Inc., 15–234) and visualized.

## Results

### hnRNP U is a novel binding partner for CENP-W

**Fig 1:**
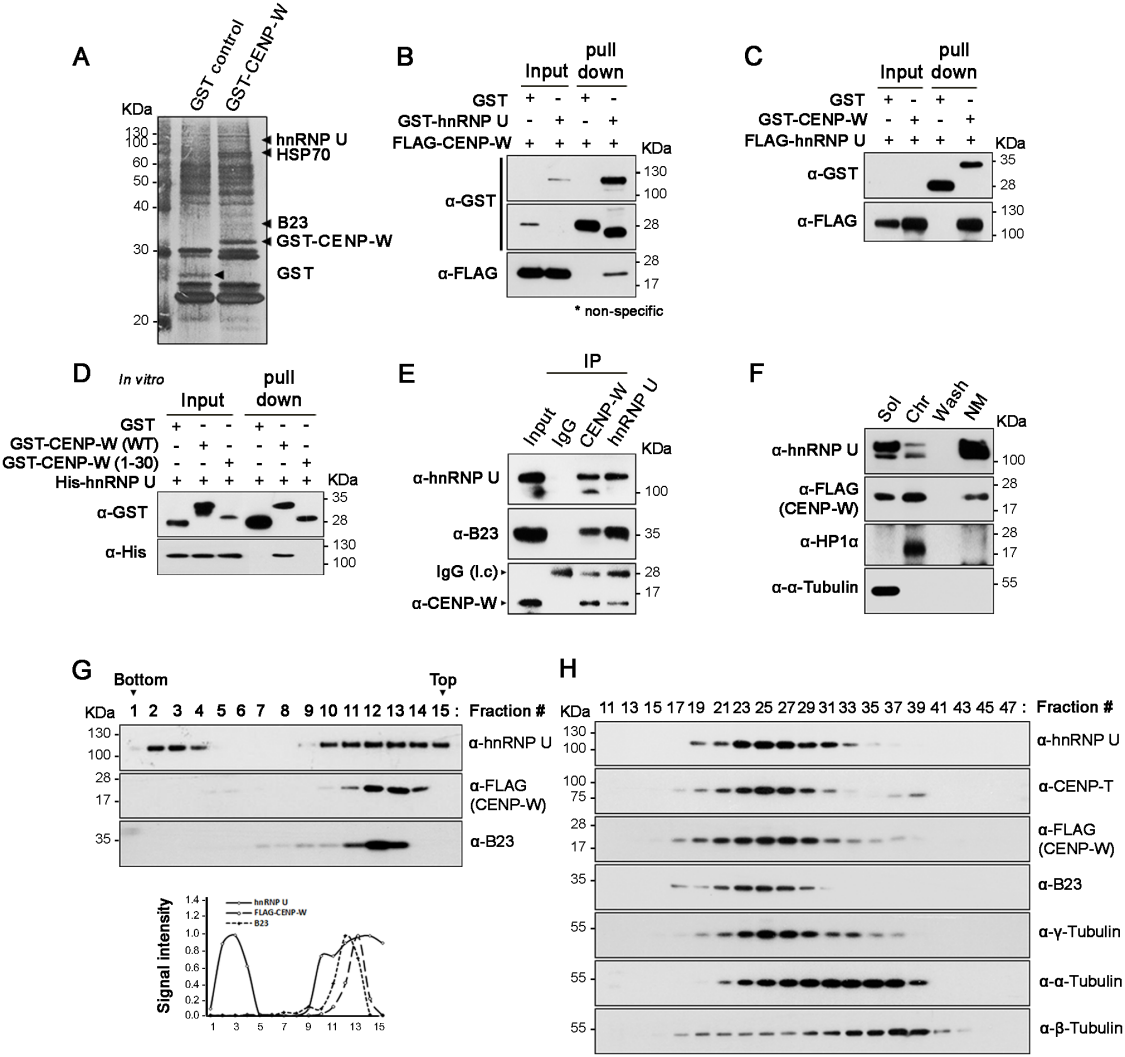
hnRNP U interacts with CENP-W. (A) After transfection of GST-CENP-W into 293T cells, GST-pulldown was performed and the CENP-W-interacting proteins were visualized using silver staining kit (Peptron) after SDS-PAGE [13]. A CENP-W-specific band was cut and analyzed by mass spectroscopy (Genomine). (B) Following transfection of GST-hnRNP U and FLAG-CENP-W, the 293T cell lysates were subjected to GST-pulldown. (C) Reciprocal interaction between GST-CENP-W and FLAG-hnRNP U using GST-pulldown. (D) In vitro binding assay; after His-hnRNP U and GST-CENP-W (either wild-type or deletion mutant (1–30 aa)) were separately expressed in *E*. *coli* Rosetta (DE3) cells using pET15b-hnRNP U and pGEX-4T-3-CENP-W, GST-pulldown was performed. (E) Binding assay at endogenous level. 293T cell lysates were used for immunoprecipitation using either anti-hnRNP U or -CENP-W antibody. Then, co-fractionated proteins were visualized using specific antibodies. (F) Nuclear matrix extraction. Cells were sequentially extracted to soluble, chromatin-enriched, and the nuclear matrix fraction following high-salt extraction method [13]. (G) HeLa-CENP-W cells were lysed and applied to the linear glycerol gradient (10–40%), and fractions collected from the bottom (fraction 1). (H) Size exclusion chromatography was performed using HeLa-CENP-W cell lysate on Sephacryl S-300 size exclusion column. Fifty 1-ml fractions were collected.

**Fig 2:**
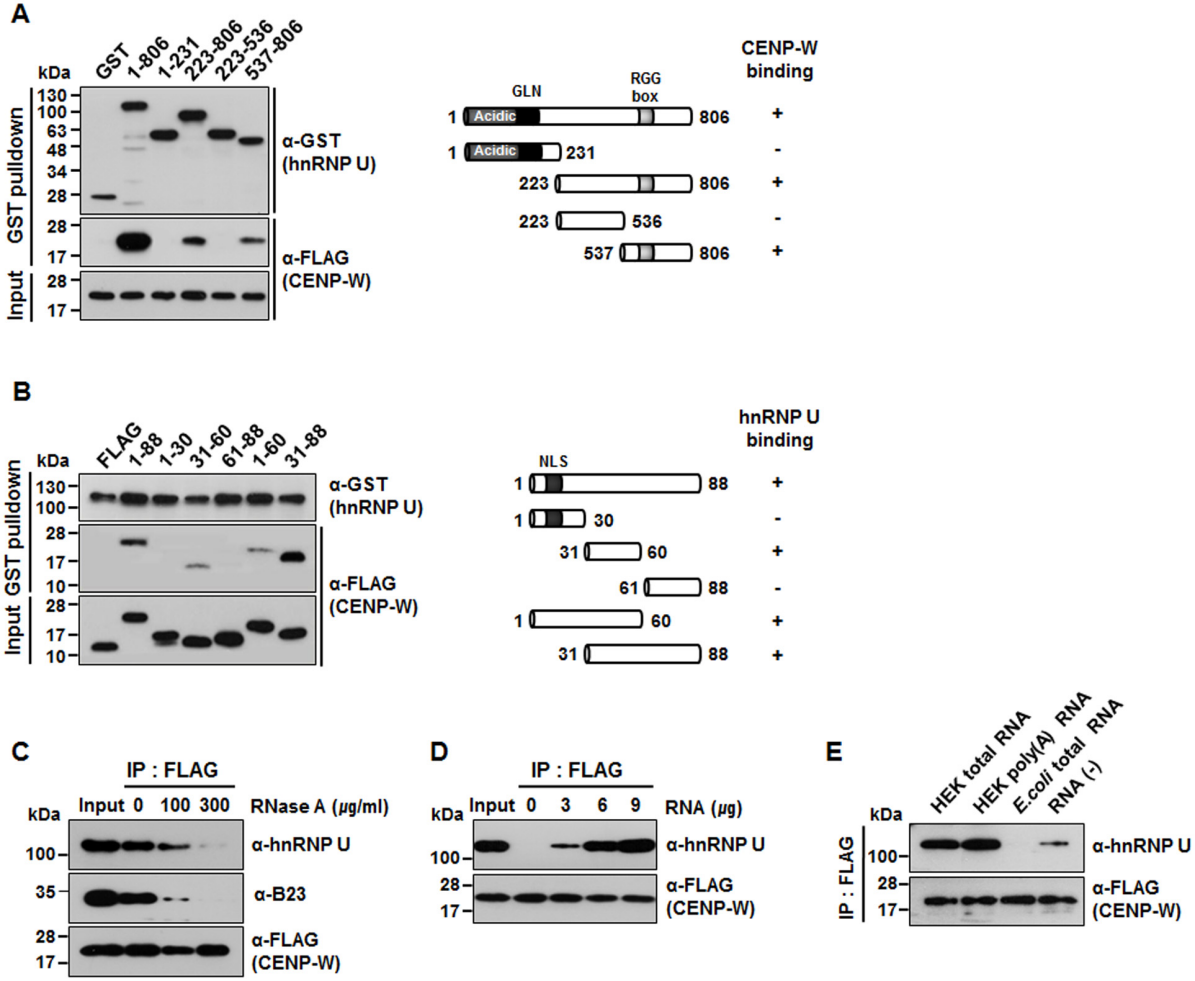
Determination of crucial domains for hnRNP U-CENP-W interaction. (A) For domain mapping of hnRNP U, GST-fused hnRNP U deletion mutants were constructed and co-transfected into 293T cells with FLAG-CENP-W. (B) GST-pulldown was performed after various FLAG-CENP-W deletion mutants were co-expressed with GST-hnRNP U. (C) Effect of RNase treatment on hnRNP U-CENP-W interaction. After HeLa-CENP-W cells were incubated with RNase A at indicated concentrations at 30°C for 20 min, immunoprecipitation was conducted with anti-FLAG antibody. (D) After cells were pre-incubated with RNase A (200 μg/mL) at 30°C for 20 min, total RNA purified from 293T cells was added before immunoprecipitation. (E) After eliminating cellular RNA with RNase A treatment (200 μg/mL), various kinds of RNAs (1 μg) were added to the samples prior to immunoprecipitation.

We have previously identified a nucleolar protein, nucleophosmin-1/B23, which interacts with CENP-W by analyzing co-isolated proteins in an affinity-binding experiment [13]. To search for more CENP-W-interacting proteins, we performed GST pulldown using 293T cell lysates transfected with either GST-CENP-W or GST control. A band that was present only in the CENP-W fraction was selected (Fig 1A). Subsequent mass spectroscopy revealed that hnRNP U is co-fractionated with CENP-W (Fig 1A). To confirm this interaction, we performed GST pulldown using ectopically expressed GST-hnRNP U and FLAG-CENP-W in 293T cells. FLAG-CENP-W was observed in the GST-hnRNP U fraction, not in that of control GST (Fig 1B). In the reciprocal experiment, FLAG-hnRNP U was co-isolated with GST-CENP-W (Fig 1C). Next, in order to demonstrate binding between CENP-W and hnRNP U using bacterially expressed recombinant proteins, we constructed pET15b-hnRNP U and pGEX-4T-3-CENP-W plasmid to express His-hnRNP U and GST-CENP-W, respectively, in *Escherichia coli*. Subsequent GST-pulldown revealed that His-hnRNP U only in the GST-CENP-W fraction (Fig 1D). Based on the domain mapping analysis (Fig 2B), an hnRNP U-nonbinding CENP-W mutant (1–30) was also tested, and no binding activity was found with this deletion mutant, supporting the specific interaction between two proteins. Finally, the specific interaction between CENP-W and hnRNP U was established by immunoprecipitation with anti-CENP-W antibody in 293T cells. Subsequent immunoblotting revealed that endogenous hnRNP U co-fractionated with CENP-W (Fig 1E, third lane). The previously identified CENPWbinding protein, nucleophosmin/B23 [13] was also examined as a control. Reciprocally, endogenous CENP-W was also found in the hnRNP U-enriched fraction (Fig 1E, fourth lane), thus confirming their endogenous interaction.

Given that both hnRNP U and CENP-W were previously found to be associated with nuclear matrix [13, 16], we examined their cellular distribution in HeLa-CENP-W cells [12]. To this end, we performed cell fractionation by high salt nuclear matrix isolation protocol [13]. The results revealed similar cellular distribution of hnRNP U and CENP-W; both were detected in the nuclear matrix as well as chromatin-associated fractions (Fig 1F). To determine whether CENP-W exists in a complex with hnRNP U, we fractionated HeLa-CENP-W cell lysates using a 10–40% glycerol gradient. Endogenous hnRNP U was found to be member of two distinct complexes, whereas the main peak for CENP-W overlapped with the small hnRNP U complex (Fig 1G). In addition, gel permeation chromatography was performed on Sephacryl S-300 column using HeLa-CENP-W lysates, and the protein distribution monitored by immunoblotting with specific antibodies. The major peaks of hnRNP U, CENP-W and CENP-T significantly overlapped at fraction 24–26 (Fig 1H), suggesting that CENP-W stably coexists with hnRNP U.

### Interaction of CENP-W with hnRNP U is RNA-dependent

In order to identify the specific hnRNP U region that interacts with CENP-W, a series of GST-fused hnRNP U domain mutants were generated. GST-pulldown was performed after the hnRNP U mutants were transfected into 293T cells along with FLAG-CENP-W. As Fig 2A demonstrates, CENP-W binding depends on the C-terminus region of hnRNP U, which contains the RGG box, a motif common to several RNA-binding proteins [17]. Reciprocally, we investigated the CENP-W domain(s) essential for binding to hnRNP U, wherein the central region of CENP-W was observed to be responsible for binding (Fig 2B) in the GST pulldown experiment.

Given that hnRNP U is an RNA-binding protein [17], and that its interaction with CENP-W depends on the RGG box (domain mapping results, this study), we next probed whether RNA participates in this interaction. HeLa-CENP-W lysates were pretreated with different concentrations of RNase A, followed by immunoprecipitation with anti-FLAG antibody. The amount of endogenous hnRNP U co-fractionated by FLAG-CENP-W was significantly reduced in proportion to the RNase A concentration (Fig 2C). Further, we determine whether purified RNA can restore the binding activity between CENP-W and hnRNP U. Toward this, we first treated the cell lysates with RNase A (200 μg/mL) to eliminate cellular RNAs, and then added total RNA isolated from 293T cells. This was followed by immunoprecipitation with specific antibodies. Indeed, interaction between CENP-W and hnRNP U was gradually restored in proportion to the RNA added (Fig 2D). This suggests that RNA plays a critical role in CENP-W-hnRNP U complex formation. We also examined the RNA type that most effectively promotes the CENP-W-hnRNP U complex formation. As shown in Fig 2E, the CENP-W-hnRNP U interaction was significantly augmented by the addition of either total RNA or mRNA isolated from 293T cells. However, total RNA from *E*. *coli* had no effect, indicating that this complex depends on the presence of eukaryotic RNA.

### Association of CENP-W with hnRNP U increases both their protein stabilities

**Fig 3:**
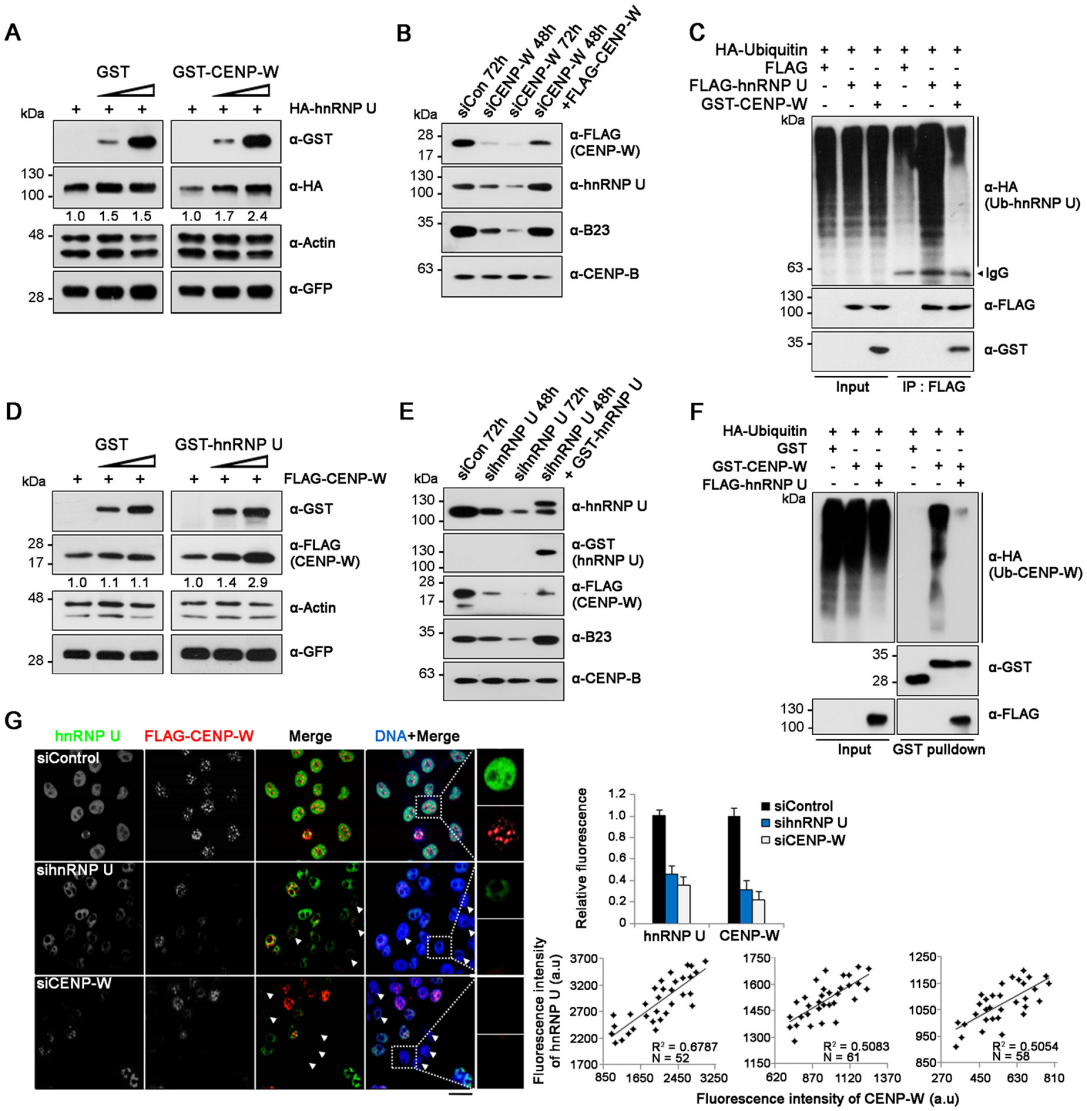
hnRNP U-CENP-W association increased their protein stability. (A) After increasing amount of GST-CENP-W plasmids were transfected to 293T cells, the protein level of co-expressed hnRNP U was analyzed by immunoblotting. (B) HeLa-CENP-W cells were incubated with CENP-W siRNAs (200 nM) for a predetermined period. For the rescue experiment, FLAG-CENP-W was transfected at 48 h after siRNA treatment and cells were incubated for another 24 h. (C) In vivo ubiquitination assay. 293T cells transfected with HA-ubiquitin and FLAG-hnRNP U were subjected to immunoprecipitation with anti-FLAG antibody. (D) 293T cells were transfected with GST-hnRNP U along with increasing amount of FLAG-CENP-W. (E) HeLa-CENP-W cells were incubated with 200 nM of hnRNP U siRNAs, followed by immunoblot analysis. If necessary, the siRNA-treated cells were transfected with GST-hnRNP U, and incubated for another 24 h. (F) GST-pulldown was performed using 293T cells transfected with HA-ubiquitin and GST-CENP-W. (G) In situ immunofluorescence staining. Following treatment with siRNAs (200 nM) specific for hnRNP U or CENP-W for 60 h, HeLa-CENP-W cells were double-immunostained with anti-hnRNP U (green) and -FLAG (red) antibodies. Scale bars = 10μm. The bar graph shows the average fluorescence intensity of hnRNP U or CENP-W in siRNA-treated cells relative to the mean fluorescence intensity of control siRNA-treated cells (N > 50 for each sample). Error bars indicate SDs. The three scatter plots are depicted and the fluorescence intensity of CENP-W (X) is plotted against that of hnRNP U in siControl-, sihnRNP U-, or siCENP-W-treated cells. The coefficient if determination (R^2^) was calculated with the linear regression (black line).

Interestingly, our double-transfection experiment revealed that the protein levels of hnRNP U and CENP-W were affected by each other. To elucidate this phenomenon, we monitored the hnRNP U level upon co-transfection of GST-CENP-W. hnRNP U levels increased gradually, corresponding to that of CENP-W (Fig 3A). GFP was also monitored as the transfection control. To support our observations, we additionally tested the endogenous hnRNP U levels in CENP-W-depleted cells. Knockdown of CENP-W using siRNAs induced a dramatic decrease in hnRNP U level, while the decreased hnRNP U level was restored by CENPW overexpression (Fig 3B). The same phenomenon was observed with the level of B23, which has been previously identified to interact with CENP-W and increase its stability. Next, we performed an in vivo ubiquitination assay to test whether degradation of hnRNP U is affected by CENP-W. The intensity of smeared bands for ubiquitin-conjugated hnRNP U was significantly decreased upon CENP-W co-transfection (Fig 3C), which suggest that CENP-W co-expression increases hnRNP U stability by inhibiting its ubiquitin-mediated degradation.

Reciprocally, we also examined the protein levels of CENP-W following co-transfection with hnRNP U. CENP-W levels increased in proportion to that of GST-hnRNP U, but remained unaffected by the GST control (Fig 3D). Next, we monitored the protein level of CENP-W in HeLa-CENP-W cells following siRNA-mediated hnRNP U knockdown. The CENP-W levels were once again decreased upon hnRNP U suppression and recovered by hnRNP U transfection (Fig 3E). Moreover, ubiquitination of CENP-W decreased significantly upon hnRNP U overexpression (Fig 3F). Finally, in situ immunostaining was also performed in HeLa-CENP-W cells following knockdown of either CENP-W or hnRNP U. We observed a significant correlation between the expression of CENP-W and hnRNP U; low CENP-W levels were observed in hnRNP U-depleted cells, and vice versa, as shown in the scatter plot (Fig 3G). Taken together, we propose that the association of CENP-W with hnRNP U mutually increases their protein stability, possibly by inhibiting their ubiquitin-proteasome-mediated degradation.

### hnRNP U is required for proper recruitment of CENP-W during mitosis

**Fig 4:**
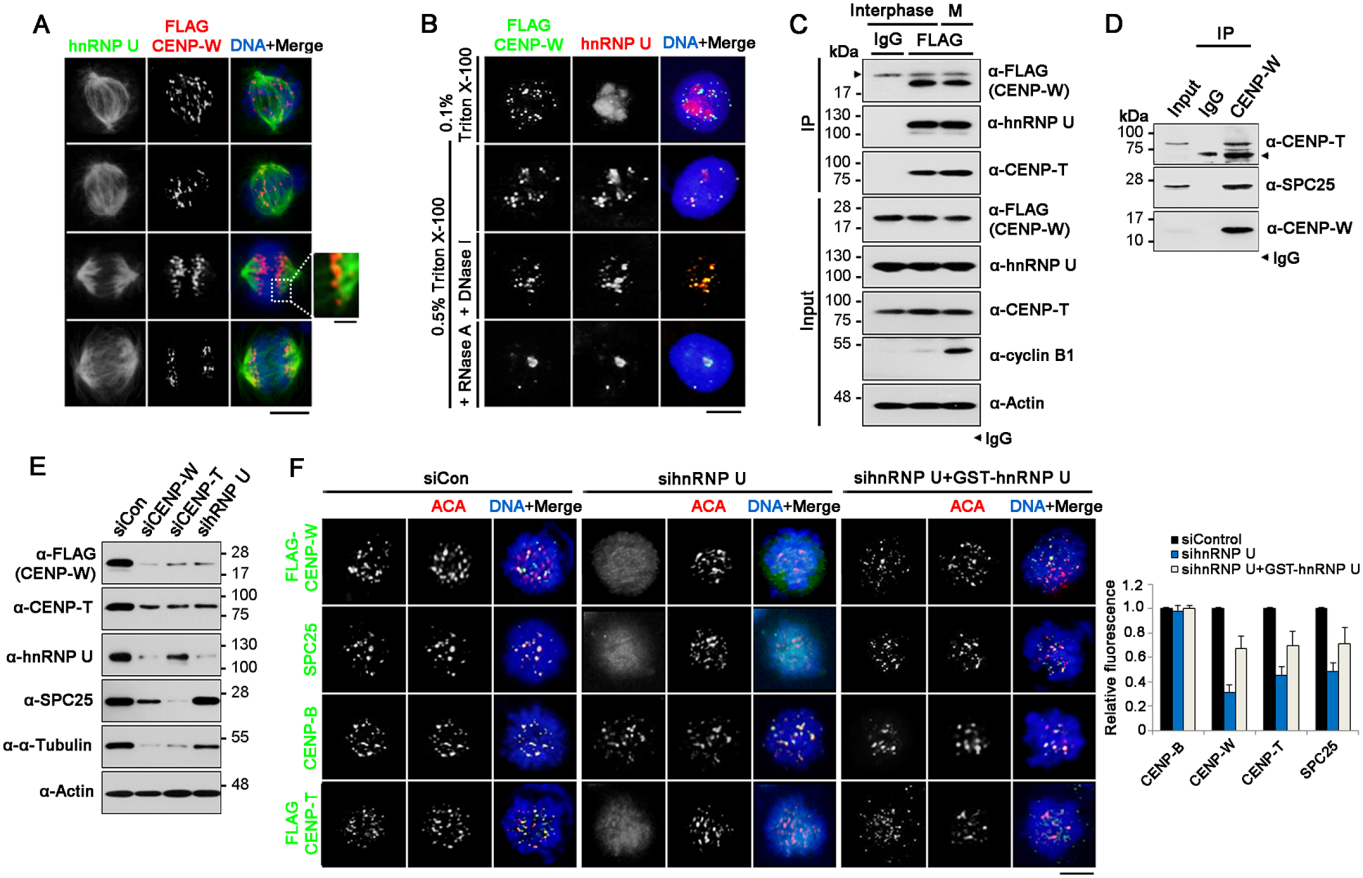
Cellular localization of hnRNP U and CENP-W. (A) Localization of CENP-W and hnRNP U during mitosis. After cells were synchronized with nocodazole (100 ng/mL) for 12h, HeLa-CENP-W cells were harvested at 30 min time-intervals and immunostained with anti-hnRNP U and -FLAG antibodies. Scale bar equals 10 μm. (B) Co-localization of hnRNP U and CENP-W during the interphase. HeLa-CENP-W cells grown in coverslips were subjected to double-immunostaining with anti-hnRNP U and -FLAG antibody (left panels). If necessary, cells were treated with either DNase I (1 unit/μL) or RNase A (200 μg/mL) in presence of 0.5% Triton X-100, followed by ammonium sulfate extraction (0.25 M). (C) After cells were synchronized with double-thymidine block, HeLa-CENP-W cells were harvested at 2 h (interphase) or 10 h (Mitosis) after release. Then, cell lysates were subjected to immunoprecipitation with an anti-FLAG antibody. (D) Interaction between CENP-W and SPC25 at endogenous level. 293T cell lysates were subjected to immunoprecipitation with anti-CENP-W antibody, and co-isolated CENP-T and SPC25 were visualized using specific antibodies. (E) After HeLa-CENP-W cells were transfected with siRNAs (200 nM) for 72 h, the protein levels of each sample were determined by immunoblotting. (F) Mislocalization of kinetochore components in hnRNP U-depleted cells. HeLa-CENP-W or HeLa-CENP-T cells were incubated with hnRNP U-specific siRNAs (200 nM) for 60 h, and further incubated with nocodazole for 12 h for cell synchronization. For the rescue, GST-hnRNP U was transfected at 48 h after siRNA treatment. At 30 min post-release, cells in mitotic prophase were immunostained with appropriate antibodies. The bar graphs show relative fluorescence signal intensities of each sample normalized by that of control siRNA-treated cells and presented as a bar graph (N > 100 for each sample). Error bars indicate SDs.

We determined whether hnRNP U and CENP-W coexist within cells. Based on the recent finding that hnRNP U is associated with microtubules [9], we examined the mitosis-specific localization of two proteins. In order to monitor cells at various mitotic stages, HeLa-CENP-W cells were arrested at early mitosis using nocodazole. After removing the nocodazole, the cells were harvested at 30 min time-intervals and each sample was double-immunostained with anti-hnRNP U and -FLAG antibodies. While CENP-W (red) was seen as centromeric dots, hnRNP U (green) was visualized over a large region from the spindle poles to the outspread microtubules (Fig 4A). Under higher magnification, cells in the metaphase revealed overlapping of the two signals, especially at the vicinity of kinetochores. Therefore, the interaction of hnRNP U and CENP-W primarily occurs at the kinetochore-microtubule interface during mitosis.

Then, to evaluate whether this interaction occurs only during mitosis, we double-stained non-synchronized HeLa-CENP-W cells using anti-hnRNP U and anti-FLAG antibodies to visualize hnRNP U and CENP-W, respectively. Under normal staining condition with 0.1% Triton X-100, specific co-localization was not clearly observed, barring a few coexisting spots. Whereas CENP-W was seen as scattered dots, hnRNP U appeared either clumped or dispersed within the nucleus (Fig 4B, first row). Upon further extraction of soluble proteins by nuclease (either DNase or RNase) digestion with 0.5% Triton X-100, their co-localization was prominently visible (Fig 4B, rows 2–4), implying that these proteins enriched in the nuclear matrix compartment. We also performed immunoprecipitation using anti-FLAG antibody with synchronized HeLa-CENP-W cells and found that CENP-W co-fractionated with hnRNP U regardless of the cell phase (Fig 4C), thus confirming steady interaction during interphase.

A recent study revealed that CENP-T interacts with SPC24/25 of the NDC80 complex in the outer kinetochore, which plays key roles in microtubule association [18]. Upon investigating whether CENP-W also interacts with NDC80 components, immunoprecipitation using either transiently expressed proteins (S1A and S1B Fig) or *E*. *coli*-expressed recombinant proteins (S1C and S1D Fig) revealed that CENP-W co-fractionated with SPC24/25. Moreover, endogenous CENP-W, CENP-T, and SPC25 were found in a complex upon immunoprecipitation with anti-CENP-W antibody in 293T cells (Fig 4D). However, since CENP-W forms a stable complex with CENP-T, and the kinetochore complex is tightly associated dense structure, we cannot exclude the possibility that the CENP-W-NDC80 interaction might be indirect. To get a closer view, we transfected HeLa-CENP-W cells with siRNAs against CENP-W, CENP-T, or hnRNP U, and monitored their levels by immunoblotting. Decrease in the CENP-W levels was similar upon depletion of either CENP-T or hnRNP U (Fig 4E). CENP-T was also similarly affected by CENP-W and hnRNP U knockdown. Interestingly, however, hnRNP U levels were more profoundly altered by CENP-W, rather than CENP-T knockdown (Fig 4E, third row). Meanwhile, decrease in SPC25 was more significant upon CENP-T, rather than CENP-W knockdown (Fig 4E, fourth row). These data demonstrate that although CENP-W/T, hnRNP U, and NDC80 are all associated either physically or functionally at the kinetochore-microtubule boundary, each component possesses a unique role in this dynamic structure.

Next, we elucidated whether hnRNP U contributes to the localization of CENP-W, CENP-T or SPC25 at the kinetochores during mitotic prophase. Toward this, HeLa-CENP-W cells or HeLa-CENP-T cells [14] were transfected with hnRNP U-specific siRNAs and further treated with nocodazole for 12 h in order to induce mitotic arrest. Then, the cells were double-immunostained with a specific antibody as well as an anti-centromere antibody (ACA) at 30 min post-release. While CENP-B distribution was not affected by hnRNP U siRNA treatment, signals for CENP-W, CENP-T and SPC25 become very weak in the hnRNP U-depleted cells, appearing as smeared dots throughout the cells (Fig 4F). These dispersed signals of all three proteins were substantially restored by hnRNP U overexpression. This indicates that depletion of hnRNP U induced mislocalization of CENP-W/T as well as NPC80 during mitotic prophase, suggesting that hnRNP U-CENP-W interaction may be important for stable kinetochore-microtubule association during mitosis.

### Dysregulated microtubule dynamics destabilize CENP-W/CENP-T

**Fig 5:**
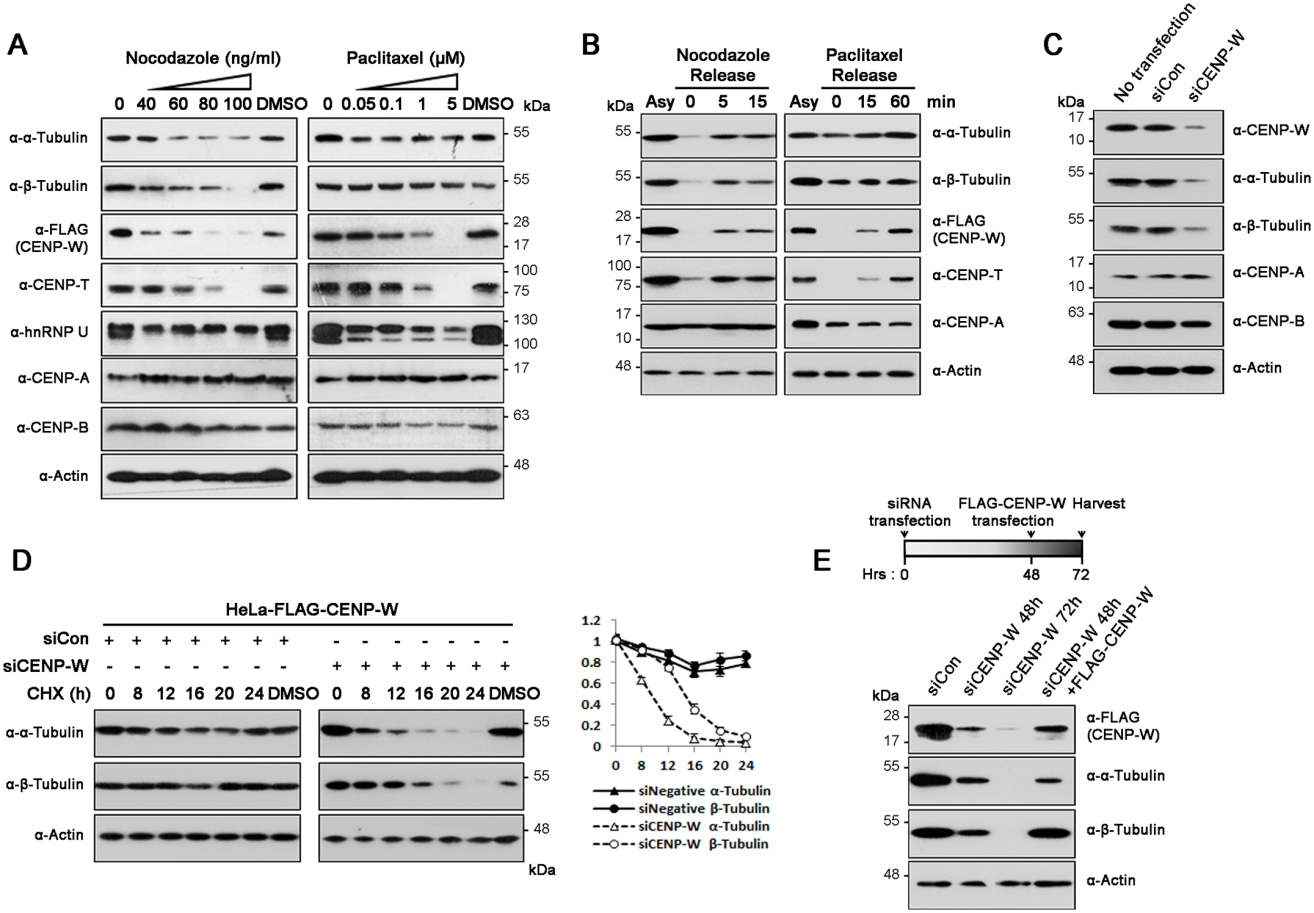
CENP-W protein level is closely related to microtubule integrity. (A) After HeLa-CENP-W cells were incubated with nocodazole or paclitaxel at indicated concentrations for 12 h, protein levels were determined by immunoblot analysis. (B) HeLa-CENP-W cells were incubated with nocodazole (100 ng/mL) or paclitaxel (5 μM) for 12 h, and harvested at indicate time points after media change. (C) HeLa cells were incubated with CENP-W-specific siRNAs (200 nM) for 72 h prior to cell harvest. (D) After HeLa-CENP-W cells were treated with siRNAs (200 nM) for 60 h, cyclohexamide (100 μg/mL) was added to the culture media. Then, cells were harvested at indicated time points and analyzed by immunoblotting. (E) Following treatment of siCENP-W for 48 h, HeLa-CENP-W cells were either continuously incubated with the siRNA for another 24 h or transfected with FLAG-CENP-W.

During the co-localization studies, we noticed that CENP-W signals weaken upon nocodazole treatment. Therefore, we next elucidated the effect of microtubule-specific drugs on kinetochore proteins. Toward this, we examined the levels of several kinetochore proteins in HeLa-CENP-W cells following incubation with increasing concentrations of either nocodazole (depolymerizes microtubules) or paclitaxel (stabilizes microtubules) for 12 h. While nocodazole dramatically decreased both α- and β-tubulin levels, paclitaxel, did not affect the stabilities of these monomers (Fig 5A). Meanwhile, the core centromere components, CENP-A and -B, showed no significant changes. However, levels of CENP-W and its known binding partner, CENP-T, were dramatically decreased by both the drugs, almost disappearing at high concentrations (Fig 5A). hnRNP U levels were also decreased, especially by paclitaxel, though this decrease did not match that of CENP-W/T. Interestingly, although the total pool of α- and β-tubulin monomers remains unaffected, significant destabilization of CENP-W and -T was observed upon paclitaxel treatment. The same phenomenon was observed when we examined the localization of kinetochore proteins following treatment with either nocodazole or paclitaxel. CENP-W and CENP-T appeared as weak and blurred dots, most of which were mislocalized outside the mitotic DNA (S2 Fig). Moreover, the CENP-W and -T levels were rapidly restored within minutes after removal of the drugs (Fig 5B). This demonstrates that microtubule homeostasis directly influences the stability of the CENP-W/T complex.

A previous study revealed that association of microtubules is less stable in hnRNP U-depleted cells [9]. We, therefore, examined the protein levels of α- and β-tubulin following CENP-W knockdown in HeLa cells. Both α- and β-tubulin proteins significantly decreased in CENP-W knockdown cells (Fig 5C). To validate these findings, we measured half-lives of α- and β-tubulin upon cyclohexamide treatment (100 μg/mL). The half-lives of both the monomers were clearly reduced in CENP-W-depleted cells versus control siRNA-treated cells (Fig 5D). Next, to elucidate the relation between α/β-tubulins and CENP-W, we pretreated cells with CENP-W-specific siRNAs for 48h, and then transfected them with FLAG-CENP-W. The protein levels of both α- and β-tubulin gradually decreased in CENP-W-depleted cells up to 72 h post transfection. Concordantly, the protein levels were restored upon CENP-W overexpression (Fig 5E), which demonstrates that stabilities of α- and β-tubulin are closed related to CENP-W. Overall, these findings indicate that cellular CENP-W/T levels and tubulin homeostasis are mutually affected and therefore, an appropriate CENP-W/T level could be required to maintain optimal microtubule dynamics.

### CENP-W possibly partially regulates microtubule stability

**Fig 6:**
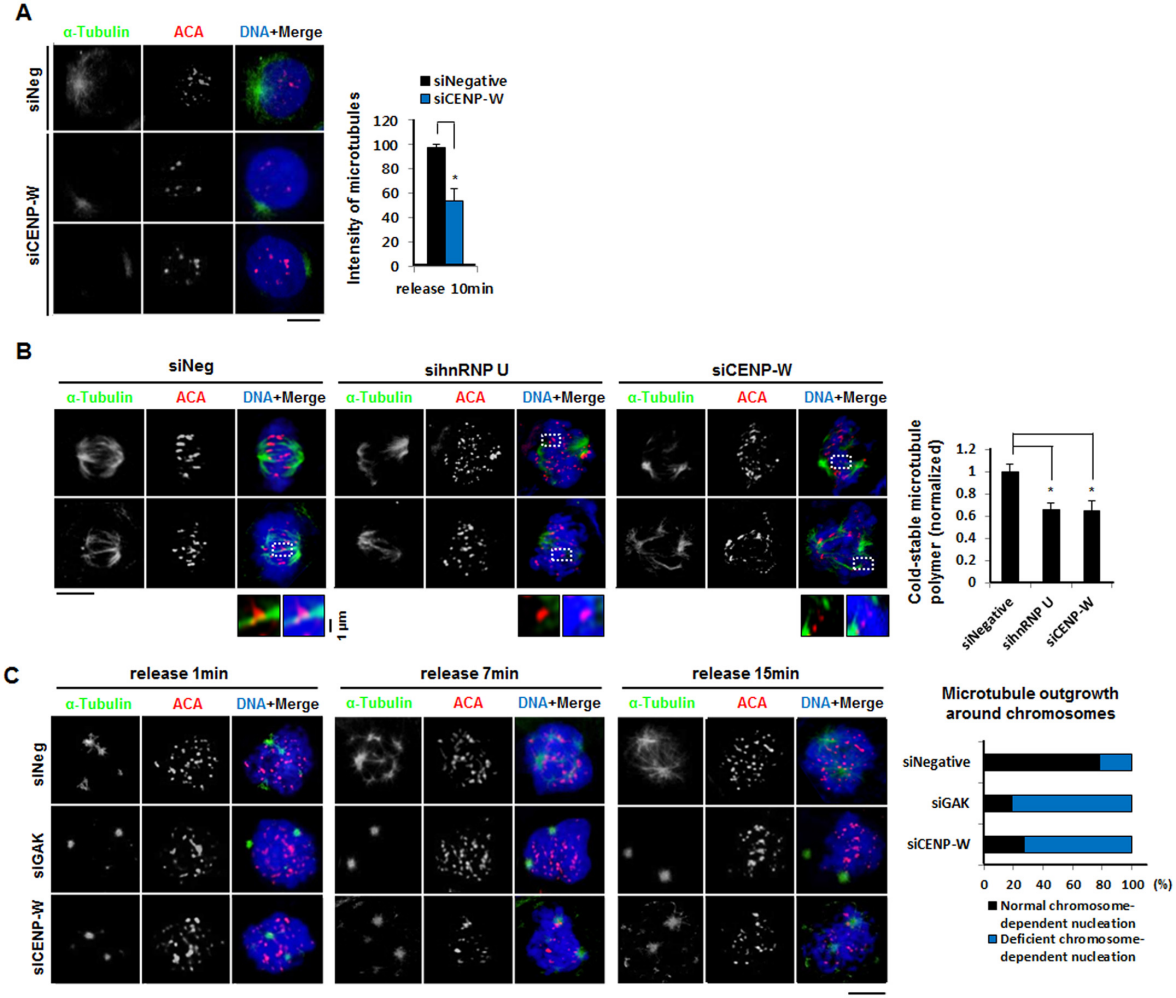
The role of CENP-W in kinetochore-microtubule interaction. (A) Microtubule regrowth assay. After pre-incubation with siRNAs for 60 h, HeLa-CENP-W cells were placed at 0°C for 30 min. Cells were then incubated at 37°C for 10 min prior to fixation. The bar graph represents microtubule intensity in cells (N > 100 for each sample). Error bars indicate SDs. *P < 0.01. (B) Cold-induced depolymerization assay. Following siRNA treatment (200 nM) for 60 h, HeLa-CENP-W cells were placed at 4°C for 10 min. After fixation, cells were immunostained with anti-α-tubulin antibody and ACA. Images of prometaphase cells were captured by Olympus IX70 fluorescence microscope. The bar graph shows microtubule intensity of each sample normalized to that of control siRNA-treated cells. (N > 100 for each sample). Error bars indicate SDs. *P < 0.01. (C) Kinetochore-derived microtubule outgrowth. Forty-eight hours post siRNA transfection, HeLa CENP-W cells were incubated with nocodazole (100 ng/mL) for 6 h. After thorough washing, cells were incubated in fresh media for short time (1, 7, or 15 min) prior to fixation. The samples were then immunostained with anti-α-tubulin and ACA. The bar graph shows the percentage of cells showing normal or deficient phenotype of chromosome-dependent nucleation after 7-min release (N > 100 for each sample).

Given that hnRNP U contributes to stable kinetochore-microtubule association [9], we next examined whether CENP-W also performs a similar function. To this end, we studied de novo microtubule polymerization in CENP-W knocked-down cells. Cells were incubated at 0°C for 30 min to eliminate preexisting microtubules. Next, the microtubules were allowed to regrow at 37°C for 10 min. Overall intensity of microtubules in CENP-W knocked-down cells was much weaker, and the length of microtubule polymers were much shorter compared with that in the control cells (Fig 6A). We further performed a cold-induced depolymerization assay. HeLa-CENP-W cells were incubated for 10 min at 4°C at 60 h post siRNA transfection, in order to induce rapid depolymerization of unstable microtubules. This was followed by immunostaining with anti-α-tubulin antibody and ACA, after which mitotic cells were visualized under a microscope. While control prometaphase cells presented relatively normal phenotypes for microtubule filaments (Fig 6B, left panels), the mitotic spindles were significantly damaged upon cold treatment in both the hnRNP U- and CENP-W-depleted cells (Fig 6B, middle and right panels). The lengths of the filaments were significantly reduced and less organized in these samples. Moreover, the number of unattached centromeres clearly increased upon knockdown of both hnRNP U and CENP-W. These results suggest that CENP-W indeed regulates kinetochore-microtubule attachment.

It is known that microtubules are nucleated at the kinetochores as well as at the centrosomes. The kinetochore-directed microtubules subsequently associated with centrosome-directed ones to form mature mitotic spindles [15]. We investigated whether CENP-W participates in kinetochore-derived microtubule nucleation. Since cyclin G-associated kinase (GAK) is required for the formation of kinetochore-directed microtubules [15], we used it as positive control. To examine de novo microtubule nucleation, cells were treated with nocodazole for 6 h and fixed at 1, 7, and 15 min post release. At the 1-min time-point, two centrosome-nucleated microtubules were clearly visible, though additional, weak kinetochore-directed microtubule sprouts were seen in the control samples (Fig 6C, first row, left panel). However, only centrosome-directed microtubules were found in both GAK- and CENP-W-depleted cells (Fig 6C, second and third rows, left panels). At the 7-min time-point, sprouts started to appear near the kinetochores in both GAK and CENP-W knockdown cells, though their signals were still weak compared to that in the controls (Fig 6C, middle panel). Even after 15 min, the kinetochore-directed microtubules in GAK- and CENP-W-depleted cells remained weaker and shorter than that in the controls (Fig 6C, right panel). Collectively, our data indicate that the kinetochore component CENP-W is intimately involved in stable kinetochore-microtubule interaction.

## Discussion

Microtubules are highly regulated units of the cytoskeleton system displaying two contradictory traits, stability and dynamics. Adequate regulation of this dual function is closely related to several essential aspects of cellular activity. Several nuclear proteins have been identified to be involved in mitotic progression by regulating mitotic spindles during mitosis. Nucleolin, a major nucleolar protein localized in the vicinity of chromosomes, is involved in kinetochore-microtubule attachment during mitosis [19]. Recently, hnRNP U has been reported to interact with nucleolin, and is required for proper spindle function [9]. In this study, we showed that CENP-W interacts with hnRNP U and functions in stable kinetochore-microtubule attachment, including chromosome-derived microtubule nucleation. Interestingly, a recent study revealed that CENP-W is required to maintain the bipolar spindle structure during mitosis via a key role in kinetochore assembly, particularly the microtubule docking region [20]. Our study demonstrating CENP-W-hnRNP U association could help to understand the dynamic, yet organized nature of the kinetochore-microtubule interface for proper mitotic progression.

Another multifunctional nucleolar phosphoprotein, nucleophosmin/B23, is also required for proper mitotic spindle function [21]. We previously demonstrated that CENP-W interacts with B23 at the nucleolar periphery during interphase. The protein stabilities of both, CENP-W and B23 are highly dependent on each other [13]. In this study, depletion of CENP-W or hnRNP U induced a dramatic decrease in B23 level (Fig 3B and 3E). Although this correlation may be an indirect phenomenon, we still propose that a close functional relationship could exist between these proteins. Their stable association during interphase may be important to maintain stability, which is required for appropriate functioning of these proteins during mitosis. Interestingly, all three proteins are known to be RNA-binding and the presence of eukaryotic RNAs is critical for their interaction (Fig 2C). Considering that centromeric non-coding RNAs play essential roles in the recruitment of kinetochore components and the formation of centromeric heterochromatin structure [22], we suggest that CENP-W and/or hnRNP U may function in the recruitment of non-coding RNAs and thereby be involved in RNA-mediated kinetochore constitution.

Although soluble pools of α/β-tubulin are considered as the source of microtubules in the cytoplasm, previous reports have revealed the presence of nuclear tubulins. Soluble tubulins are thought to dynamically shuttle between the cytoplasm and nucleus [23]. Aberrant nuclear accumulation of tubulin is often observed in cancer cell lines or transformed cells [24], suggesting a specific role of nuclear tubulins in cancer. Nuclear tubulin is thought to interfere with important nuclear functions, interacting with H1 and core histones [25]. Consistently, CENP-W/T exhibits histone-like function at the centromere [26] and microtubule-disturbing drugs rapidly decreased CENP-W/T protein stability in this study (Fig 5A and 5B). Thus, we speculate that CENP-W/T may not only interact with mitotic spindles during mitosis, but also dynamically associate with soluble tubulin monomers during interphase. Taken that CENP-W was originally identified as an overexpressed gene in human tumor biopsies, we suggest that CENP-W could also be a useful target to investigate a cancer-related role of nuclear tubulins.

Kinetochores are dynamic proteinaceous multicomplexes, conventionally subdivided into the inner and outer region based on their architecture captured by electron microscopy [27]. The inner kinetochore consists of repetitive centromeric DNA and its associated proteins, which more constitutively localize in this region [28]. The outer kinetochore, on the other hand, consists of relatively more temporary centromeric residents including key players in the association of spindle microtubules during mitosis [4]. However, recent studies have also implicated components residing in the inner kinetochore, such as CENP-C and CENP-T, in this process [18, 29]. This indicates that the inner kinetochore components not only regulate truthful formation of centromeric chromatin, but also that of proper outer kinetochore complexes and subsequent attachment of microtubules. This study introduces another inner kinetochore component, CENP-W, as a participant in the formation of functional outer kinetochore complex and kinetochore-microtubule attachment. This potentiates the recently proposed role for the inner kinetochore components as active players in the microtubule attachment in mitotic cells.

## Supporting Information

S1 Fig CENP-W may form a complex with NDC80. (A) After 293T cells were transfected with HA-SPC25, GST-CENP-W, and FLAG-hnRNP U, immunoprecipitation was performed using anti-HA antibody. (B) Immunoprecipitation was performed using anti-HA antibody using ectopically expressed HA-SPC24, GST-CENP-W, and FLAG-hnRNP U. (C) Interaction between recombinant proteins. After His-HA-SPC25 and GST-CENP-W were expressed in *E*.*coli*, the bacterial lysates were used for GST-pulldown. (D) After *E*.*coli* lysates were obtained, in vitro interaction between His-CENP-T, His-HA-SPC25, and GST-CENP-W was examined by immunoprecipitation using anti-HA antibody.
(TIF)

S2 Fig Distribution of centromere proteins after drug treatment. HeLa-CENP-W cells cultured in coverslips were treated with nocodazole (100 ng/mL) or paclitaxel (1 μM) for 12 h, and fixed at 10 min after release. Then, double-immunostainning was performed with anti-α-tubulin antibody along with specific antibodies for CENP-A, -B, -C, or -T. CENP-W was examined using anti-FLAG antibody. Scale bars = 10 μm.
(TIF)
