# Supplementary figures and images for: Centromere Protein (CENP)-W Interacts with Heterogeneous Nuclear Ribonucleoprotein (hnRNP) U and May Contribute to Kinetochore-Microtubule Attachment in Mitotic Cells
Source: PLoS One. 2016 Feb 16;11(2):e0149127. doi: 10.1371/journal.pone.0149127 (PMC4755543; doi:10.1371/journal.pone.0149127)

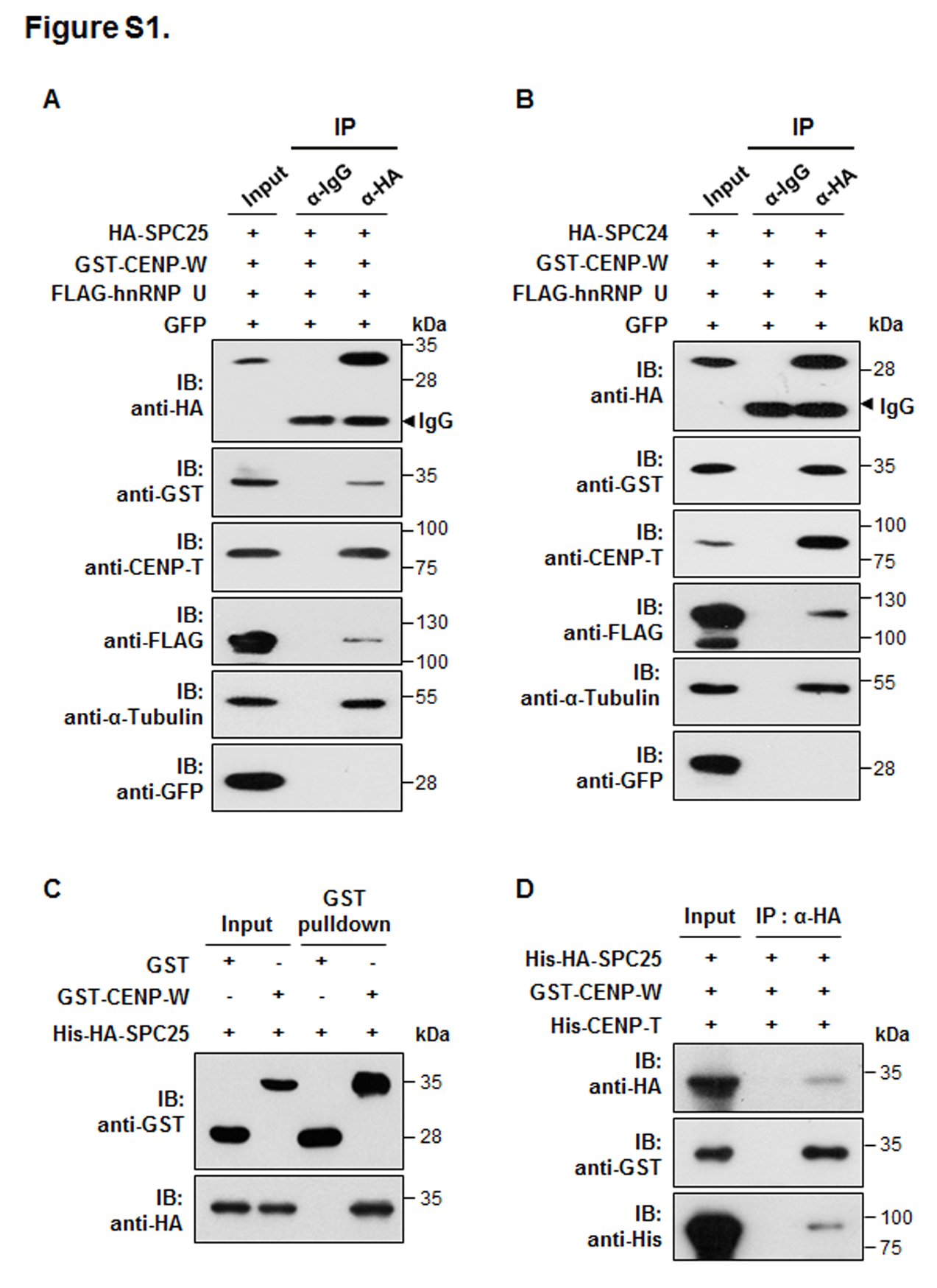

Supplement: S1 Fig — (A) After 293T cells were transfected with HA-SPC25, GST-CENP-W, and FLAG-hnRNP U, immunoprecipitation was performed using anti-HA antibody. (B) Immunoprecipitation was performed using anti-HA antibody using ectopically expressed HA-SPC24, GST-CENP-W, and FLAG-hnRNP U. (C) Interaction between recombinant proteins. After His-HA-SPC25 and GST-CENP-W were expressed in E.coli, the bacterial lysates were used for GST-pulldown. (D) After E.coli lysates were obtained, in vitro interaction between His-CENP-T, His-HA-SPC25, and GST-CENP-W was examined by immunoprecipitation using anti-HA antibody. (TIF) [file pone.0149127.s001.tif]

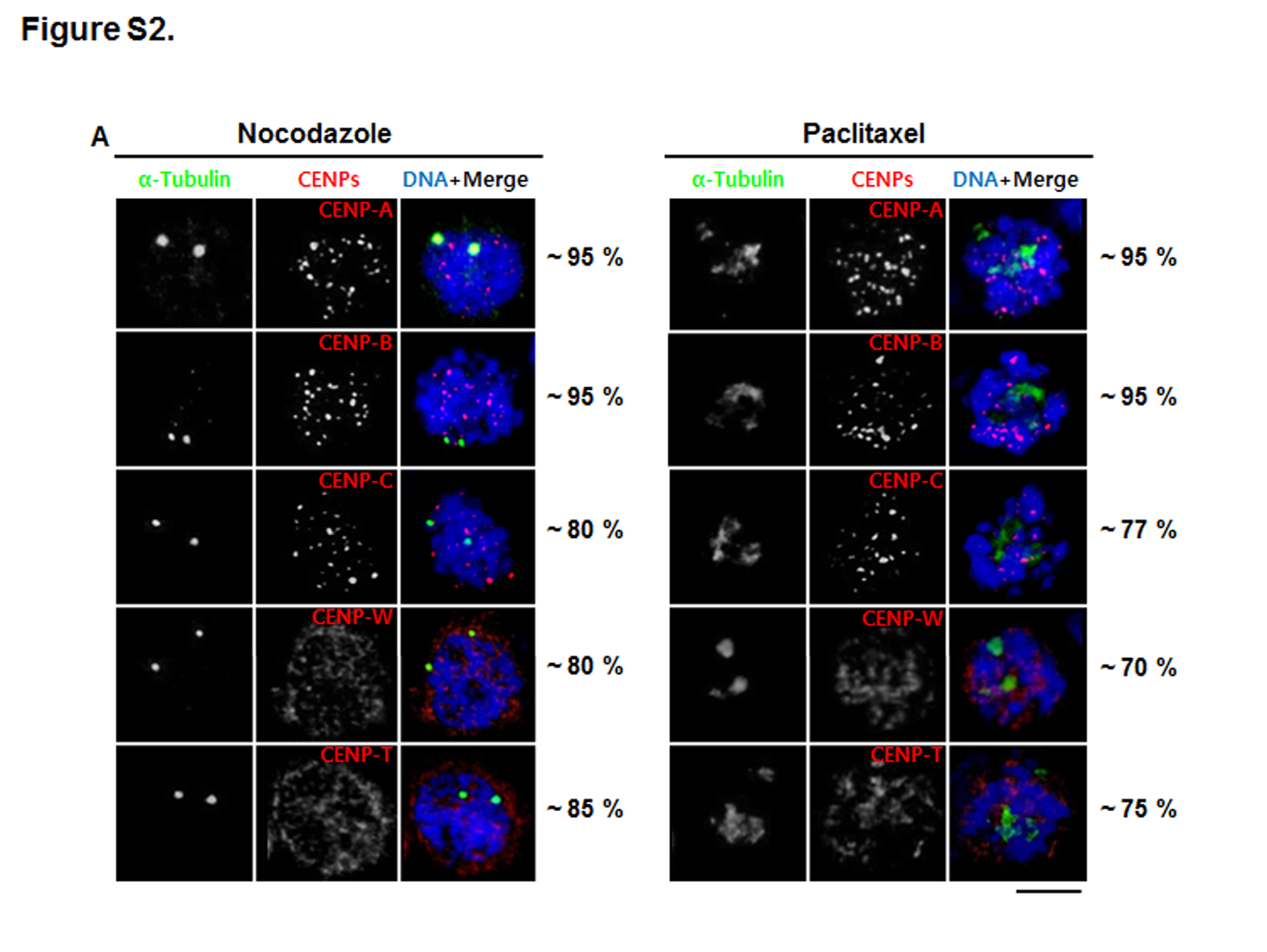

Supplement: S2 Fig — HeLa-CENP-W cells cultured in coverslips were treated with nocodazole (100 ng/mL) or paclitaxel (1 μM) for 12 h, and fixed at 10 min after release. Then, double-immunostainning was performed with anti-α-tubulin antibody along with specific antibodies for CENP-A, -B, -C, or -T. CENP-W was examined using anti-FLAG antibody. Scale bars = 10 μm. (TIF) [file pone.0149127.s002.tif]
